# Identification of a criminal by DNA typing in a rape case in Rio de Janeiro, Brazil

**DOI:** 10.1590/S1516-31802002000300004

**Published:** 2002-05-02

**Authors:** Andréa Carla de Souza Góes, Dayse Aparecida da Silva, Cristiane Santana Domingues, João Marreiro Sobrinho, Elizeu Fagundes de Carvalho

**Keywords:** Sexual abuse, Forensic DNA, STR, VNTR, Paternity, Abuso sexual, DNA forense, STR, VNTR, Paternidade

## Abstract

**CONTEXT:**

Human DNA identification is a powerful tool for paternity cases as well as for criminal investigation, in which biological evidence is typed after collection from crime scenes and for the identification of human remains.

**OBJECTIVE:**

Identification of a criminal in a rape case with 4 suspects using STR and VNTR DNA analysis.

**TYPE OF STUDY:**

Forensic DNA analysis.

**SETTING:**

DNA Diagnostic Laboratory, Universidade Estadual do Rio de Janeiro, Brazil.

**PARTICIPANTS:**

Blood from 4 suspects and the victim, and skin from the fetus.

**PROCEDURES:**

Polymerase chain reaction (PCR) and restriction fragment length polymorphism (RFLP).

**RESULTS:**

Three of the suspects were excluded and one of them was identified as the biological father of the fetus after typing with CTT and FFv Multiplexes. Complementary DNA typing at 3 VNTR loci was also carried out.

**CONCLUSIONS:**

After typing four suspects using 6 STR loci, one of them was identified as the biological father of the fetus. In order to significantly enhance the Combined Paternity Index (PI), complementary DNA typing in 3 VNTR loci was carried out. The included suspect was found to be the biological father with a PI of 412,860 (Probability of Paternity: 99.9997%).

## RESUMO

**CONTEXTO:**

A identificação humana por DNA é uma ferramenta poderosa para casos de paternidade, assim como investigação criminal pela tipagem de evidências biológicas coletadas em cenas de crime e identificação de restos mortais.

**OBJETIVO:**

Identificação de criminoso em caso de estupro com quatro suspeitos utilizando análise de STR e VNTR no DNA.

**TIPO DE ESTUDO:**

Análise de DNA forense.

**LOCAL:**

Laboratório de Diagnósticos por DNA, Universidade do Estado do Rio de Janeiro, Brasil.

**PARTICIPANTES:**

Sangue de quatro suspeitos e vítima e pele do feto.

**PROCEDIMENTOS:**

Reação em cadeia da polimerase (PCR) e polimorfismo de tamanho de fragmentos de restrição (RFLP).

**RESULTADOS:**

Três suspeitos foram excluídos e um suspeito foi incluído como pai biológico do feto após tipagem com os Multiplexes CTT e FFv. Um teste complementar com 3 *loci* VNTR também foi realizado.

**CONCLUSÃO:**

Após a tipagem de quatro suspeitos em 6 *loci* STR, um deles foi incluído como o pai biológico do feto. Com o objetivo de aumentar o Índice de Paternidade Combinado (PI), um teste complementar com 3 *loci* VNTR foi realizado. O suspeito foi incluído como pai biológico apresentando Índice de Paternidade de 412.860 (probabilidade de paternidade: 99,9997%).

## INTRODUCTION

Since 1985, DNA has been shown to be a powerful tool in paternity cases, as well as for criminal investigation.^1^ This is accomplished by extracting DNA from any biological material, such as blood, saliva, skin, muscle, hair, sperm, teeth, bone, etc.^2,3,4^ Afterwards, comparison of individuals typed at polymorphic DNA regions can be done as "in tandem" repeated sequences. This kind of comparison can be achieved because one component of each of the 23 pairs of chromosomes characteristic of the human being is inherited from the biological father and the other from the biological mother. In the light of this, human identification can be accomplished by DNA comparison between an individual and the alleged biological mother, father or other possible genetically related persons such as grandmothers, grandfathers, brothers, sisters or uncles. In the same way, the DNA profile of biological samples collected at a crime scene can be compared to that from a suspect or his relatives. The scientific knowledge gained in this area has enabled DNA typing of human remains and biological evidence to become a valid technique for human identification.

In this context, polymorphic regions within DNA, called VNTR (variable number of tandem repeats), have been successfully used for DNA typing purposes for more than 10 years.^5^ However, as the analysis of VNTR loci requires large amounts of DNA, this methodology is not so efficient for typing degraded DNA and samples with very small amounts of DNA, such as the DNA prepared from biological samples collected from the environment. On the other hand, STR (short tandem repeats) analysis is a simple methodology that works even with poor and degraded DNA. As a disadvantage, it is known that a STR *locus* is not as polymorphic as a VNTR one.^6^

In this report, we describe the usefulness of both methodologies for typing samples collected from a four-month-old fetus conceived in a rape case. The victim, a 14-year-old girl with a genetic disorder (Down's Syndrome), was raped in Rio de Janeiro, Brazil, in 1998. The crime was only notified some months later when the pregnancy became evident, and therefore DNA sperm typing was not carried out to identify the rapist from among the suspects. The victim had obtained judicial authorization for an abortion and, in order to identify the biological father of the fetus, we typed the aborted fetus, its mother (the victim) and four suspects in the rape case. We combined the STR and VNTR analyses to increase the Paternity Index.

## METHODS

***DNA extraction*** - 50 μL of blood from the victim and suspects, and skin tissue from the fetus (10-20 mm^2^), were incubated for 15-18 hours in 500 μL lysis buffer (10 mM TrisCl pH 7.5; 1 mM EDTA; 50 mM NaCl; 2% SDS) containing 0.3 mg/ml proteinase K, at 56 °C. The DNA was extracted using phenol/chloroform/isoamyl alcohol (25:24:1). Each DNA preparation was resuspended in 20 μL TE buffer (10 mM Tris-Cl, 1 mM EDTA). In order to estimate DNA integrity, 1 μL samples were applied to 0.8% agarose gel, with electrophoresis at 100V for 15 minutes and staining with ethidium bromide (0.5 μg/ml). DNA quantification was carried out by measuring optical densities at 260 nm.

***PCR reaction*** - 6 STR *loci* were analyzed using the FFv and CTT Multiplex silver-stained systems from Promega Corporation. To a 25 μL final reaction volume, 5 ng of template DNA, 2.5 μL STR 10 buffer, 2.5 μL CTT or FFv primer and 0.75 U Taq polymerase were added. The PCR reactions were performed in a Perkin-Elmer 9600 thermocycler with initial denaturation for 2 min at 96 °C followed by 10 cycles of 1 min at 94 °C, 1 min at 60 °C, 1.5 min at 70 °C and 20 cycles of 1 min at 90 °C, 1 min at 60 °C and 1.5 min at 70 °C. The amplification products were analyzed on a 2% agararose gel.

***STR allele analysis*** - The amplification products were resolved on a 4% polyacrylamide gel (7 M urea; 4% acrylamide-bisacrylamide solution; 0.5 TBE), using SA32 Sequencing Gel Electrophoresis Apparatus (Life Technologies). The gel was pre-run for 30 minutes at 1500V, 80W, 40 mA, in 0.5 × TBE buffer prior to sample loading. The PCR products were mixed with loading buffer and denatured at 95 °C for 2 min. The gel was run for 90 min under the same conditions as above and with silver staining.^7^

***DNA digestion with restriction enzyme*** - 500 ng of genomic DNA were digested with 1U of Hae III (Gibco-BRL), at 37 °C for 12-18 hours. The restriction fragments were precipitated by adding ammonium acetate to 2.5 M final and 2 volumes of ethanol. After incubation at −20 °C for 2 hours, the DNA was recovered by centrifugation in a microfuge (maximum speed, for 10 minutes) and resuspended in 20 μL loading buffer (10 mM Tris-Cl pH 7.5; 1 mM EDTA; 20% Ficoll; 0.02% BPB; 0.02% XC-FF solution).

***Electrophoresis and DNA transfer*** - The fragments were separated on 0.8% agarose gel with 1 × TBE buffer for 24 hours at 20V. The gel was denatured using alkali treatment and transferred to nylon membrane by the Southern method.^8^

**Hybridization** - Sequential hybridization was developed as recommended by the manufacturers, using alkaline-phosphatase chemiluminescence labeled probes AC415, CEB42 and TBQ7.

***Statistical analysis*** - A data bank from the population of the State of Rio de Janeiro was utilized.^9,10^

## RESULTS

The silver-stained polyacrylamide gel (Figures 2 and 3) showed that three of the suspects were excluded and only suspect # 3 was included as the biological father of the fetus after typing with CTT and FFv Multiplexes. The typed alleles at the 6 STR *loci* for each suspect are summarized in Table 1. Suspect # 1 was excluded because of the TPOX, F13A01 and vWA *loci*. Suspect # 2 was excluded because of 5 of the 6 STR *loci*, whereas suspect # 4 was excluded because of 4 *loci*. In addition, in order to increase the PI for suspect # 3, we also typed the victim, fetus and suspect # 3, using 3 VNTR *loci*. The results from the VNTR analysis are summarized in Table 1. A cumulative paternity index of 412,860 was obtained by combining the STR and VNTR analysis. The probability of paternity was 99.9997%.

**Figure 1:**
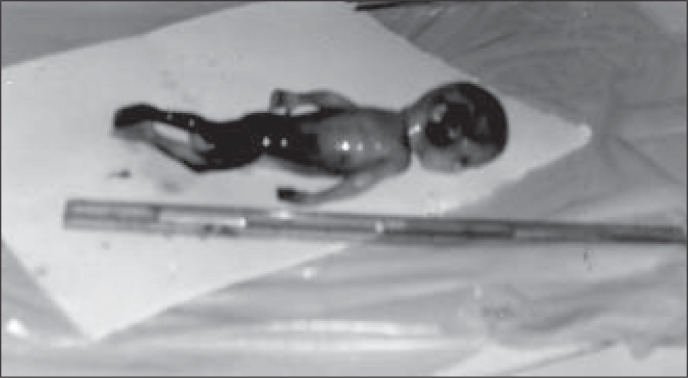
Aborted fetus obtained after judicial authorization.

**Figure 2:**
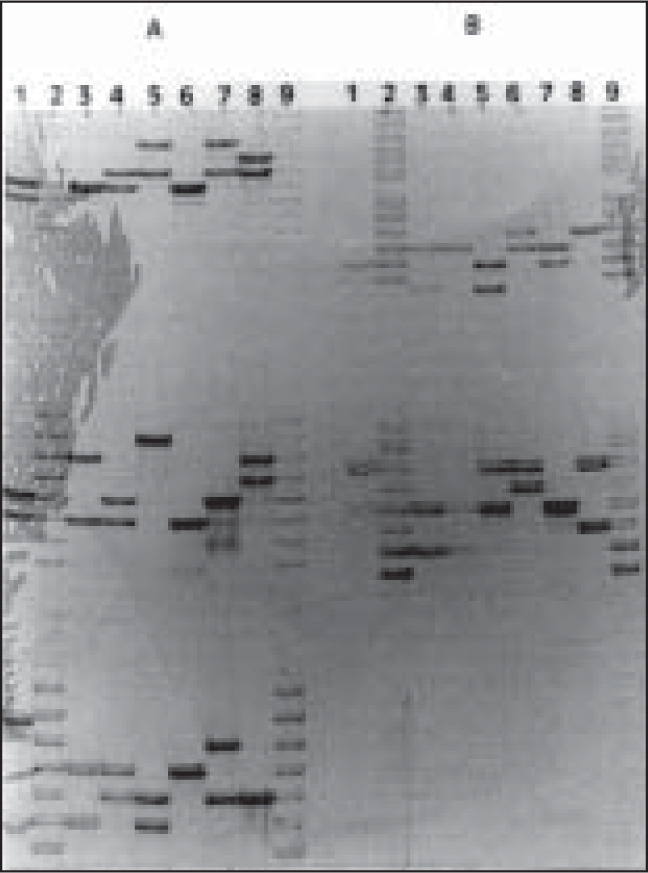
4% silver-stained polyacrylamide gel showing CTT, F13A01 and FESFPS typing of victim, fetus and suspects. PANEL A: CTT (CSF1PO, TPOX and TH01) typing. PANEL B: F13A01 and FESFPS typing. 1- positive control; 2- ladder; 3- victim (mother); 4- fetus; 5- Suspect # 1; 6- Suspect # 2; 7- Suspect # 3; 8- Suspect # 4; 9- ladder

**Figure 3:**
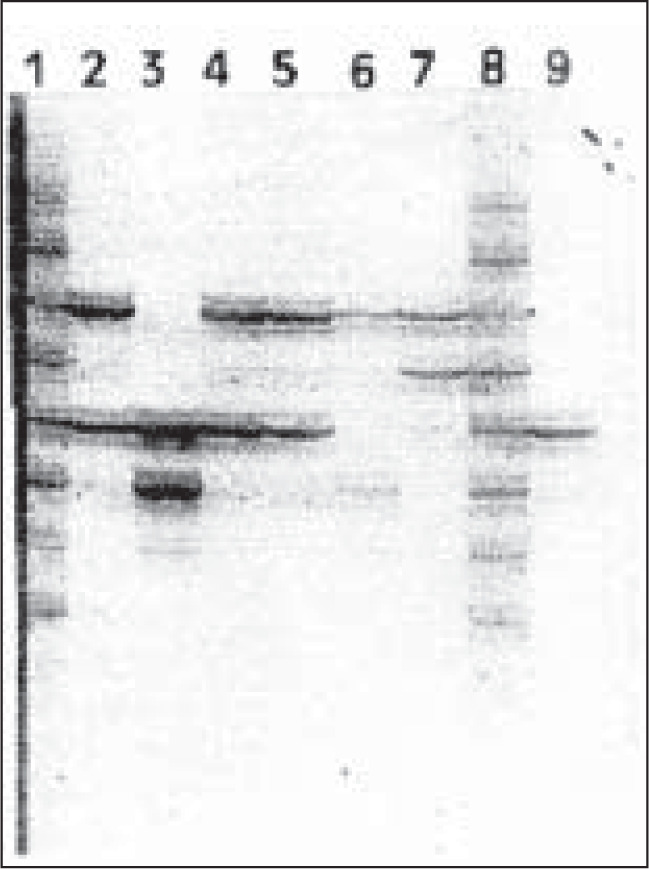
6% silver-stained polyacrylamide gel showing vWA typing of victim, fetus and suspects. 1- ladder; 2- Suspect # 4; 3- Suspect # 3; 4- Suspect # 2; 5- Suspect # 1; 6- fetus; 7- victim (mother); 8- ladder; 9- positive control.

**Table 1:** STR genetic profiles of victim, fetus and suspects (CSF1PO, TPOX, TH01, F13A01, FESFPS, vWA) and VNTR genetic profiles of victim, fetus and suspect # 3 (D7S467, D8S358, D10S28). The frequency of the obligatory paternal allele in the fetus that is simultaneously observed in the suspect #3 and the Paternity Index is shown for each *locus*

Locus	Victim (mother)	Fetus		Suspect #1	Suspect #2	Suspect #3	Suspect #4	AF	PI
CSF1PO	10	10	(m)	11	10	11	11	0.33	1.51
	10	11	(p)	13	10	13	12		
TPOX	8	8	(m)	12	8	9	10	0.08	12.5
	11	9	(p)	12	8	9	11		
THO1	6	7	(p)	6	8	7	7	0.18	2.77
	8	8	(m)	7	8	9	7		
F13A01	3.2	6	(p)	3.2	6	5	7	0.32	1.56
	6	6	(m)	5	7	6	7		
FESFPS	8	8	(m/p)	10	11	10	9	0.25	3.38
	10	10	(m/p)	12	12	10	12		
VWA	17	15	(p)	16	16	15	16	0.13	3.84
	18	18	(m)	18	18	16	18		
D7S467	5920	4549	(m)	NT	NT	4549	NT	0.16	3.12
	4549	4549	(p)			2760			
D8S358	5080	5080	(m)	NT	NT	4240	NT	0.05	10.0
	1270	3200	(p)			3200			
D10S28	5100	2920	(p)	NT	NT	2920	NT	0.04	12.5
	1420	1420	(m)			1840			
		**Combined Paternity Index**						**412,860**	

*The suspect's alleles coincident with the obligatory paternal alleles are underlined; AF - Fetus and suspect #3 coincident allele frequency; PI - Paternity Index; NT - Not typed; m - maternal allele; p- obligatory paternal allele.*

## DISCUSSION

Nowadays, in rape cases, routine vaginal swabs can be taken from the victims to carry out DNA analysis. The genetic profiles and even the number of criminals involved in sexual crimes can be determined from the sperm DNA typing. In this case, the crime was not reported to the authorities immediately after the rape occurred. As sperm was not collected to compare the spermatozoa profile to those of the four suspects, the fetus DNA profile was compared to them. This procedure would be expected to indicate whether at least one of the suspects was the biological father of the fetus. In view of this, all four suspects were tested to identify the biological father of the aborted fetus (Figure 1). We utilized as strategy a combination of VNTR and STR analysis to carry out the Paternity testing. The paternal alleles of the fetus were found in the genomic material of suspect #3 at all examined *loci* as would be expected for a father and his child.

## CONCLUSIONS

Suspect # 3 was found to be the biological father of the fetus with a Combined Paternity Index of 412,860 and a Probability of Paternity of 99.9997%.
